# Risk factors of nosocomial infection after cardiac surgery in children with congenital heart disease

**DOI:** 10.1186/s12879-020-4769-6

**Published:** 2020-01-21

**Authors:** Xindi Yu, Maolin Chen, Xu Liu, Yiwei Chen, Zedong Hao, Haibo Zhang, Wei Wang

**Affiliations:** 1 Department of Cardiothoracic Surgery, Shanghai Children’s Medical Center, School of Medicine, Shanghai Jiao Tong University, 1678 Dongfang Road, Pudong district, Shanghai, China; 2 Shanghai Synyi Medical Technology Co., Ltd, Shanghai, China

**Keywords:** Nosocomial infection, Cardiac surgery, Children

## Abstract

**Background:**

The aim of our study was to analyze the risk factors of nosocomial infection after cardiac surgery in children with congenital heart disease (CHD).

**Methods:**

We performed a retrospective cohort study, and children with CHD who underwent open-heart surgeries at Shanghai Children’s Medical Center from January 1, 2012 to December 31, 2018 were included. The baseline characteristics of these patients of different ages, including neonates (0–1 months old), infants (1–12 months old) and children (1–10 years old), were analyzed, and the association of risk factors with postoperative nosocomial infection were assessed.

**Results:**

A total of 11,651 subjects were included in the study. The overall nosocomial infection rate was 10.8%. Nosocomial infection rates in neonates, infants, and children with congenital heart disease were 32.9, 15.4, and 5.2%, respectively. Multivariate logistic regression analysis found age (OR 0798, 95%CI: 0.769–0.829; *P* < 0.001), STS risk grade (OR 1.267, 95%CI: 1.159–1.385; P < 0.001), body mass index (BMI) <5th percentile (OR 1.295, 95%CI: 1.023–1.639; *P* = 0.032), BMI >95th percentile (OR 0.792, 95%CI: 0.647–0.969; *P* = 0.023), cardiopulmonary bypass (CPB) time (OR 1.008, 95%CI: 1.003–1.012; *P* < 0.001) and aortic clamping time (OR 1.009, 1.002–1.015; *P* = 0.008) were significantly associated with nosocomial infection in CHD infants. After adjusted for confounding factors, we found STS risk grade (OR 1.38, 95%CI: 1.167–1.633; *P* < 0.001), BMI < 5th percentile (OR 1.934, 95%CI: 1.377–2.715; *P* < 0.001), CPB time (OR 1.018, 95%CI: 1.015–1.022; P < 0.001), lymphocyte/WBC ratio<cut off value (OR 3.818, 95%CI: 1.529–9.533; *P* = 0.004) and AST>cut off value (OR 1.546, 95%CI: 1.119–2.136; *P* = 0.008) were significantly associated with nosocomial infection in CHD children.

**Conclusion:**

Our study suggested STS risk grade, BMI, CPB duration, low lymphocyte/WBC or high neutrophil/WBC ratio were independently associated with nosocomial infection in CHD infant and children after cardiac surgery.

## Background

In-hospital infections have an adverse effect on the clinical outcomes of pediatric patients after thoracotomy, which can cause morbidity, mortality, prolonged hospital stay [1–4]. The prevalence of nosocomial infections in children with open-heart surgery remains high. Among patients who admitted into the ICU, 50% or more of the patients were affected by nosocomial infections, compared with about 5 to 15% of inpatients in the conventional ward [5]. In developing countries, the severity of nosocomial infections is still underestimated, mainly due to lack of good surveillance system which requires expertise and resources [6].

In-hospital infections after cardiac surgery in patients with congenital heart disease are affected by many factors. A prospective cohort study found that diabetes mellitus and obesity are associated with surgical site infection in valve surgery, and diabetes mellitus and reoperation for bleeding are associated with surgical site infection in coronary revascularization [7]. Younger age and ventilator or ECMO use at time of heart transplant are attribute to bacterial infections of pediatric patients [8].

A 4-year survey found that age < 2 months, congenital malformations, post-operative complications, and open-chest procedure are associated with nosocomial infections in pediatric cardiovascular surgery patients [9]. Surgery-related risk factors include age, longer preoperative hospitalizations, higher American Society of Anesthesiologist (ASA) score, preoperative ventilation, longer duration of surgical procedures, blood transfusions, continuation of antimicrobial prophylaxis more than 48 h, longer ICU time and longer hospitalization time, open chest, surgical risk grade, and duration of central line placement are associated with hospital-acquired infections in pediatric cardiac surgical patients [10]. The study founded that risk factors for nosocomial infection after neonatal cardiac surgery include lower Apgar score, higher incidence of other congenital malformations, longer hospital stay, central venous catheter indwelling time > 14 days, mechanical ventilation time > 7 days, blood product transfusion > 5 times [11].

Previous studies on risk factors for nosocomial infection in children with CHD have often focused on a specific age group, and few studies have detailed analysis of risk factors for nosocomial infections after CHD in children of different ages, especially in China. Therefore, the aim of this study was to explore the risk factors of post-operative in-hospital infection in children with CHD classified according to age. The study also used nomogram to analyze the discrimination ability of these risk factors for the post-operative infection. The discrimination ability of the nomograms was evaluated through the area under the (ROC) curve (AUC).

## Methods

### Patient and study design

Children with CHD who underwent cardiac surgery at Shanghai Children’s Medical Center from January 1, 2012 to December 31, 2018 were included in this retrospective cohort study. For patients with multiple surgery records within 30 days, only the last open-heart surgery was included and the previous surgery records were considered as operation history. The inclusion criteria include patient with CHD, age ≤ 10 years old. The exclusion criteria include surgery without CPB, infection within preoperative 30 days, preoperative ventilator support, and no complete blood count (CBC) within 48 h before CPB, deaths of uninfected patients after surgery, postoperative infection occurred within 2 days of admission. Demographic characteristics and pre-operation test data were extracted from a clinical database. The study was approved by the Ethical Committee of Shanghai Children’s Medical Center. All children with congenital heart disease were divided into neonates (0–1 months old), infants (1–12 months old) and children (1–10 years old) according to age. We analyzed the in-hospital infection rate of all CHD children after cardiac surgery. The baseline characteristics of these patients of different ages were analyzed, and the association of risk factors with postoperative nosocomial infection were assessed.

### Definition and variables

Nosocomial infection was defined according to draft version of the hospital infection diagnosis standard of Chinese Ministry of Health [12]. The variables of interest in the study included preoperative demographic data of the patient, laboratory test data after admission, surgical related variables. All these study data were obtained from the hospital’s electronic medical record. All laboratory testing was done at Shanghai Children’s Medical Center. The cut-offs and selection criteria for laboratory tests were listed in Additional file 1: Table S1. Moreover, the STS risk grade was defined as ordinal data; an increase in the STS risk grade can indicate the risk of nosocomial infection [13] .

### Statistical analysis

Continuous variables were described as Mean ± SD or Median (Range) according to their distributions and categorical data were presented as frequency (%). For between group comparison, T tests or Mann-Whitney U tests were used for continuous variables and Chi-squared tests or Fisher’s exact tests for categorical data, as appropriate.

To identify risk factors for nosocomial infection, univariate logistic regression was used first and multiple logistic regression were applied for significant variables (i.e. *P* < 0.05) in the univariate analysis to select the significant risk factors.

Then, nomograms were established for the risk predictive models for nosocomial infection, taking the whole samples for infant subgroup and child subgroup as derivation cohorts, respectively. Finally, cross validation was conducted as internal validation.

All tests were two-sided and *p* < 0.05 were regarded as statistically significant.

## Results

A total of 11,651 subjects were included in the study. Among them, there are 85 newborns, 6183 infants, and 5383 children. Nosocomial infection rates in neonates, infants, and children with congenital heart disease were 32.9,15.4, and 5.2%, respectively. The overall nosocomial infection rate was 10.8%. There were 3739 patients who underwent surgery between 2012 and 2014, and the incidence of nosocomial infection was 12%. There were 7912 patients who underwent surgery between 2015 and 2018, the hospital infection rate was 10.3%. Among 1259 cases of nosocomial infection, there were 989 ventilator-associated pneumonia, 188 urinary system infections, 71 systemic infections, 10 catheter-related bacteremia, and 1 upper respiratory tract infection (Table 1). The interval from the end of surgery to infection of catheter-related bacteremia was 193.3 h (137.9, 267.0), the time interval for ventilator-associated pneumonia, urinary tract infection, upper respiratory infection and systemic Infection were 24.5 h (21.0, 48.8), 96.2 h (87.2, 120.7), 21.9 h (21.9, 21.9) and 46.6 h (14.0, 98.5). The deaths were found in ventilator-associated pneumonia patients (3.03%), urinary tract infection patients (1.06%), and systemic infection (4.23). Postoperative length of stays was 8–17.5 d in all infection patients (Table 1).

**Table 1:** Comparison of clinical outcomes in patients with different infection types

Infection type	Number	Interval from the end of surgery to infection (hour) Median (IQR)	Deaths (mortality)	Postoperative length of stays (day) Median (IQR)
Catheter-related bacteremia	10	193.3 (137.9, 267.0)	0 (0%)	17.5 (11.0, 42.0)
Ventilator-associated pneumonia	989	24.5 (21.0, 48.8)	30 (3.03%)	11 (8.0, 14.0)
Urinary system infection	188	96.2 (87.2, 120.7)	2 (1.06%)	12 (10.0, 15.0)
Upper respiratory tract infection	1	21.9	0 (0%)	8
Systemic infection	71	46.6 (14.0, 98.5)	3 (4.23%)	13 (7.0, 16.0)
Total	1259	40.2 (21.9, 74.9)	35 (2.78%)	12 (9.0, 15.0)

### Newborns

#### Baseline characteristics and postoperative outcomes of newborns with and without nosocomial infection

Of all 85 neonatal CHD patients, there were 28 nosocomial infections and 91 controls. There were no significant differences in length of hospital stay and mortality after operation between nosocomial infection patients and control (*P* = 0.124, *P* = 0.329) (Table 2). General characteristics were similar for nosocomial infection and control in CHD newborns. Significant differences were found in age (19.5d vs. 14d, *P* = 0.039), Neutrophil count (*P* = 0.026) between of nosocomial infection and control (Additional file 1: Table S1).

**Table 2:** Postoperative hospital stays and outcome comparison between nosocomial infection neonates and controls

		Nosocomial infection	Control	*P* value
*Neonates*	N	28	57	
Length of hospital stay (d)	Median (IQR)	12 (9,17.5)	11 (9,13)	0.124
Outcomes	Mortality	1 (3.57%)	0 (0%)	0.329
	No death	27 (96.43%)	57 (100%)	
*Infants*	N	952	5231	
Length of stay (d)	Median (IQR)	11 (9,14)	7 (6,10)	< 0.001
Outcomes	Mortality	20 (2.1%)	0 (0%)	< 0.001
	No death	932 (97.9%)	5231 (100%)	
*Children*	N	279	5104	
Length of stay (d)	Median (IQR)	12 (8,16)	6 (6,8)	< 0.001
Outcomes	Mortality	14 (5.02%)	0 (0%)	< 0.001
	No death	265 (94.98%)	5104 (100%)	

### Infants

#### Baseline characteristics and postoperative outcomes of infants with and without nosocomial infection

Among 6183 CHD infants (median age 188d, range: 122-250d), there were 952 nosocomial infections, and 5231controls. Among baseline characteristics, CPB time (69 min vs. 51 min, *P* < 0.001) and aortic clamping time (42 min vs. 31 min, *P* < 0.001) of nosocomial infections infant was significantly higher than control group, but age (138d vs. 196d, *P* < 0.001) was significantly younger. BMI, STS risk grade, delayed sternal closure, serum creatinine level, lymphocyte count, neutrophil count, lymphocyte/white blood cell (WBC) ratio and neutrophil/WBC ratio were all significantly different between nosocomial infection and control in CHD newborns (Table 3). The length of hospital stay and mortality were significantly different between nosocomial infection and control after cardiac surgery (both *P* < 0.001) (Table 2).

**Table 3:** Baseline characteristics of postoperative infection infants and control

Parameter		Postoperative Infection (*n* = 952)	Control (*n* = 5231)	Total (*n* = 6183)	*P* value
Age (days)	Median (IQR)	138 (87.5208)	196 (130,255)	188 (122,250)	< 0.001^
CPB time (min)	Median (IQR)	69 (51,98)	51 (40,70)	54 (41,74)	< 0.001^
Aortic clamping time (min)	Median (IQR)	42 (28,61)	31 (23,43)	32 (23,46)	< 0.001^
Gender	MALE	564 (59.24%)	2978 (56.93%)	3542 (57.29%)	0.184#
Preterm birth	YES	7 (0.74%)	45 (0.86%)	52 (0.84%)	0.698#
History of cardiac surgery	YES	13 (1.37%)	58 (1.11%)	71 (1.15%)	0.494#
BMI	< 5th percentile	148 (16.95%)	1040 (21.24%)	1188 (20.59%)	0.013#
	5th~95th percentile	508 (58.19%)	2744 (56.05%)	3252 (56.37%)	
	> 95th percentile	217 (24.86%)	1112 (22.71%)	1329 (23.04%)	
STS risk grade	1	366 (38.45%)	3150 (60.22%)	3516 (56.87%)	< 0.001^
	2	310 (32.56%)	1443 (27.59%)	1753 (28.35%)	
	3	158 (16.6%)	332 (6.35%)	490 (7.92%)	
	4	116 (12.18%)	304 (5.81%)	420 (6.79%)	
	5	2 (0.21%)	2 (0.04%)	4 (0.06%)	
Delayed sternal closure	YES	9 (0.95%)	8 (0.15%)	17 (0.27%)	< 0.001##
ALT	< cut off value	19 (2.3%)	79 (1.6%)	98 (1.7%)	0.224#
	Normal	737 (89.23%)	4473 (90.84%)	5210 (90.61%)	
	>cut off value	70 (8.47%)	372 (7.55%)	442 (7.69%)	
AST	Normal	316 (38.16%)	1880 (38.03%)	2196 (38.05%)	0.943#
	>cut off value	512 (61.84%)	3063 (61.97%)	3575 (61.95%)	
ALP	Normal	819 (99.15%)	4910 (99.39%)	5729 (99.36%)	0.424#
	>cut off value	7 (0.85%)	30 (0.61%)	37 (0.64%)	
Serum creatinine	< cut off value	7 (0.85%)	37 (0.75%)	44 (0.77%)	0.013##
	Normal	814 (98.79%)	4888 (99.23%)	5702 (99.17%)	
	>cut off value	3 (0.36%)	1 (0.02%)	4 (0.07%)	
WBC counts	< cut off value	18 (1.89%)	55 (1.05%)	73 (1.18%)	0.075#
	Normal	898 (94.33%)	4956 (94.74%)	5854 (94.68%)	
	>cut off value	36 (3.78%)	220 (4.21%)	256 (4.14%)	
Lymphocyte count	< cut off value	32 (3.36%)	153 (2.92%)	185 (2.99%)	0.002#
	Normal	260 (27.31%)	1166 (22.29%)	1426 (23.06%)	
	>cut off value	660 (69.33%)	3912 (74.78%)	4572 (73.94%)	
Neutrophil count	< cut off value	228 (23.95%)	1357 (25.94%)	1585 (25.63%)	0.022#
	Normal	677 (71.11%)	3703 (70.79%)	4380 (70.84%)	
	>cut off value	47 (4.94%)	171 (3.27%)	218 (3.53%)	
Lymphocytes/WBC	< cut off value	76 (7.98%)	170 (3.25%)	246 (3.98%)	< 0.001#
	Normal	331 (34.77%)	1435 (27.43%)	1766 (28.56%)	
	>cut off value	545 (57.25%)	3626 (69.32%)	4171 (67.46%)	
Neutrophils/WBC	< cut off value	599 (62.92%)	3831 (73.24%)	4430 (71.65%)	< 0.001#
	Normal	184 (19.33%)	902 (17.24%)	1086 (17.56%)	
	>cut off value	169 (17.75%)	498 (9.52%)	667 (10.79%)	

Note:^ Mann-Whitney U test; ^^ T test; # Chi-square test; ## Fisher exact method

*CPB* cardiopulmonary bypass, *BMI* body mass index, STS risk grade: Society of Thoracic Surgeons risk grade, *ALT* alanine transaminase, *AST* aspartate aminotransferase, *ALP* alkaline phosphatase, *WBC* white blood cell

#### Univariate and multivariate analysis of risk factors for nosocomial infection in CHD infants

Significant risk factors in the univariate analysis associated with nosocomial infection were age, STS risk grade, delayed sternal closure, BMI < 5th percentile, CPB time, aortic clamping time, lymphocyte count >cut off value, lymphocyte/WBC ratio, neutrophil count >cut off value, neutrophil/WBC ratio and serum creatinine >cut off value (Table 4).

**Table 4:** Univariate and multivariate logistic regression analysis of risk factors for postoperative infection in infants with congenital heart disease

Parameter		Univariate analysis		Multivariate analysis	
		OR (95% CI)	*P*-value	OR (95% CI)	*P* value
Age (days)	Unit = 30	0.812 (0.789,0.835)	< 0.001	0.798 (0.769,0.829)	< 0.001
Gender	Female	1.000			
	Male	1.1 (0.956,1.265)	0.184		
Preterm birth	No	1.000			
	Yes	0.854 (0.384,1.899)	0.698		
History of cardiac surgery	No	1.000			
	Yes	1.235 (0.674,2.262)	0.495		
STS risk grade	Unit = 1	1.625 (1.517,1.74)	< 0.001	1.267 (1.159,1.385)	< 0.001
Delayed sternal closure	No	1.000			
	Yes	6.235 (2.4,16.203)	< 0.001		
BMI	5th–95th percentile	1.000			
	< 5th percentile	0.769 (0.632,0.936)	0.009	1.295 (1.023,1.639)	0.032
	> 95th percentile	1.054 (0.886,1.254)	0.552	0.792 (0.647,0.969)	0.023
CPB time	Unit = 1	1.017 (1.015,1.019)	< 0.001	1.008 (1.003,1.012)	< 0.001
Aortic clamping time	Unit = 1	1.023 (1.02,1.026)	< 0.001	1.009 (1.002,1.015)	0.008
Lymphocyte count	Normal	1.000			
	< cut off value	0.938 (0.626,1.405)	0.756		
	>cut off value	0.757 (0.646,0.886)	0.001		
Lymphocytes /WBC	Normal	1.000			
	< cut off value	1.938 (1.442,2.605)	< 0.001		
	>cut off value	0.652 (0.561,0.757)	< 0.001		
Neutrophil count	Normal	1.000			
	< cut off value	0.919 (0.781,1.081)	0.308		
	>cut off value	1.503 (1.078,2.097)	0.016		
Neutrophil /WBC	Normal	1.000			
	< cut off value	0.766 (0.64,0.918)	0.004	0.838 (0.676,1.039)	0.107
	>cut off value	1.664 (1.314,2.106)	< 0.001	1.264 (0.939,1.7)	0.122
ALT	Normal	1.000			
	< cut off value	1.461 (0.88,2.425)	0.143		
	>cut off value	1.142 (0.874,1.492)	0.329		
AST	Normal	1.000			
	>cut off value	0.994 (0.855,1.157)	0.943		
ALP	Normal	1.000			
	>cut off value	1.4 (0.613,3.196)	0.425		
Serum creatinine	Normal	1.000			
	< cut off value	1.136 (0.505,2.557)	0.758		
	>cut off value	18.008 (1.871,173.3)	0.012		

Note: *CPB* cardiopulmonary bypass, *BMI* body mass index, STS risk grade: Society of Thoracic Surgeons risk grade, *ALT* alanine transaminase, *AST* aspartate aminotransferase, *ALP* alkaline phosphatase, *WBC* white blood cell

After adjusted confounding factors, the study found age (OR 0.798, 95%CI: 0.769–0.829; *P* < 0.001), STS risk grade (OR 1.267, 95%CI: 1.159–1.385; P < 0.001), BMI <5th percentile (OR 1295, 95%CI: 1.023–1.639; *P* = 0.032), BMI >95th percentile (OR 0.792, 95%CI: 0.647–0.969; *P* = 0.023), CPB time (OR 1.008, 95%CI: 1.003–1.012; P < 0.001), aortic clamping time (OR 1.009, 95%CI: 1.002–1.015; *P* = 0.008) were significantly associated with nosocomial infection in CHD infants (Table 4).

#### Nomograms predicting nosocomial infection risk of CHD infant after cardiac surgery

**Fig. 1:**
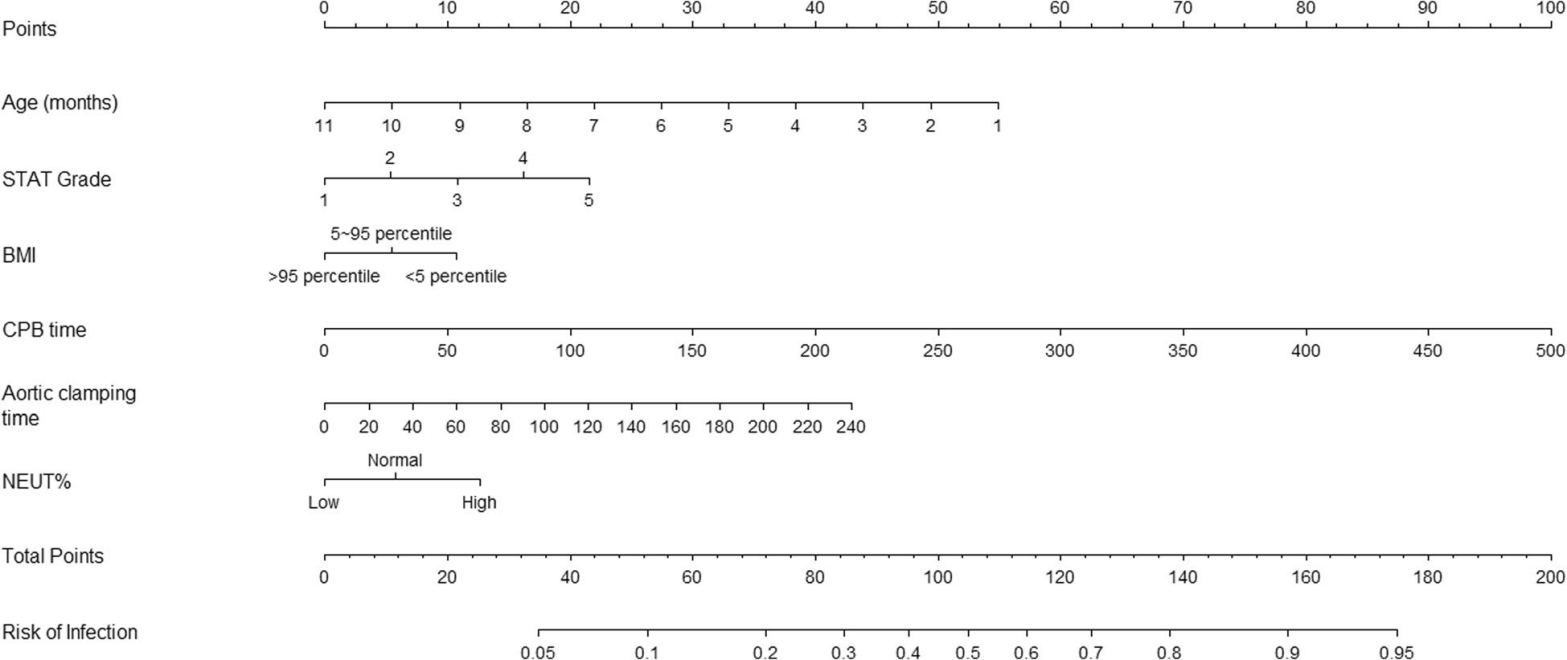
Nomograms predicting nosocomial infection risk of CHD infant after cardiac surgery

**Fig. 2:**
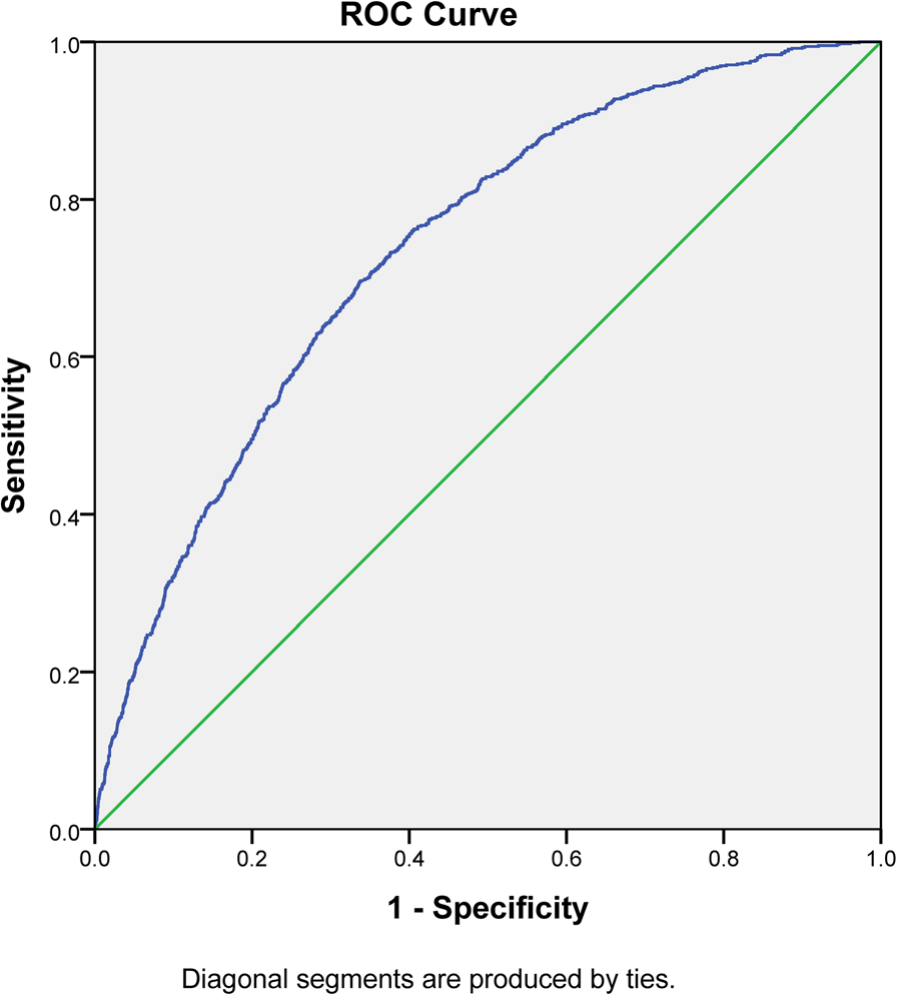
The area under the curve of nomograms predicting nosocomial infection risk of CHD infant after cardiac surgery

Nosocomial infection probability can be estimated with the nomograms (Fig. 1). In order to calculate the probability of nosocomial infection after heart surgery in infants with congenital heart disease, each parameter has a corresponding score on the point axis, and the sum of the scores is plotted on the “total point” axis. The probability of nosocomial infection is the value at a vertical line from corresponding total points. The area under the curve (AUC) of nomograms predicting nosocomial infection risk of CHD infant after cardiac surgery was 0.738 (95% CI: 0.721–0.755, *P* < 0.001). After cross validation, AUC of nomograms was 0.730 (Fig. 2).

### Children

#### Baseline characteristics and postoperative outcomes of children with and without nosocomial infection

Among 5383 CHD children (median age 929 d, range: 545–1458 d), there were 279 nosocomial infections and 5104 controls. Few characteristics were similar for nosocomial infection and control patients. Nosocomial infection CHD children had longer CPB time (88 min vs. 48 min, *P* < 0.001) and aortic clamping time (52 min vs. 28 min, *P* < 0.001). Characteristics of nosocomial infection CHD children including proportion of patients with a history of cardiac surgery, BMI, STS risk grade, proportion of patients with delayed sternal closure, abnormal ALT, AST, WBC counts, lymphocyte counts, neutrophil count, lymphocyte/WBC ratio and neutrophil/WBC ratio were all significantly different with those of control in CHD children (Table 5). The length of hospital stay and mortality were significantly different between nosocomial infection and control after cardiac surgery (both P < 0.001) (Table 2).

**Table 5:** Baseline characteristics of postoperative infection children and control

Parameter		Postoperative infection (*n* = 279)	Control (*n* = 5104)	Total (*n* = 5383)	*P* value
Age (days)	Median (IQR)	832 (489,1386)	933 (548,1459.5)	929 (545,1458)	0.057^
CPB time (min)	Median (IQR)	88 (66,125)	48 (36,72)	49 (36,75)	< 0.001^
Aortic clamping time (min)	Median (IQR)	52 (39,76)	28 (19,44)	29 (19,46)	< 0.001^
Gender	MALE	152 (54.48%)	2828 (55.41%)	2980 (55.36%)	0.762#
Preterm birth	YES	2 (0.72%)	37 (0.72%)	39 (0.72%)	> 0.999##
History of cardiac surgery	YES	82 (29.39%)	673 (13.19%)	755 (14.03%)	< 0.001#
BMI	< 5th percentile	77 (29.84%)	748 (15.83%)	825 (16.56%)	< 0.001#
	5th–95th percentile	174 (67.44%)	3734 (79.03%)	3908 (78.43%)	
	> 95th percentile	7 (2.71%)	243 (5.14%)	250 (5.02%)	
STS risk grade	1	61 (21.86%)	3064 (60.03%)	3125 (58.05%)	< 0.001^
	2	144 (51.61%)	1497 (29.33%)	1641 (30.48%)	
	3	37 (13.26%)	356 (6.97%)	393 (7.3%)	
	4	34 (12.19%)	182 (3.57%)	216 (4.01%)	
	5	3 (1.08%)	5 (0.1%)	8 (0.15%)	
Delayed sternal closure	YES	4 (1.43%)	8 (0.16%)	12 (0.22%)	0.003##
ALT	< cut off value	19 (8.23%)	315 (6.53%)	334 (6.61%)	< 0.001#
	Normal	200 (86.58%)	4435 (91.97%)	4635 (91.73%)	
	>cut off value	12 (5.19%)	72 (1.49%)	84 (1.66%)	
AST	Normal	157 (65.15%)	3825 (78.13%)	3982 (77.52%)	< 0.001#
	>cut off value	84 (34.85%)	1071 (21.88%)	1155 (22.48%)	
ALP	Normal	240 (99.59%)	4860 (99.31%)	5100 (99.32%)	> 0.999##
	>cut off value	1 (0.41%)	34 (0.69%)	35 (0.68%)	
Serum creatinine	< cut off value	0 (0%)	4 (0.08%)	4 (0.08%)	0.145##
	Normal	239 (99.58%)	4885 (99.88%)	5124 (99.86%)	
	>cut off value	1 (0.42%)	2 (0.04%)	3 (0.06%)	
WBC counts	< cut off value	4 (1.43%)	30 (0.59%)	34 (0.63%)	0.008#
	Normal	263 (94.27%)	4970 (97.37%)	5233 (97.21%)	
	>cut off value	12 (4.3%)	104 (2.04%)	116 (2.15%)	
Lymphocyte count	< cut off value	7 (2.51%)	26 (0.51%)	33 (0.61%)	< 0.001#
	Normal	101 (36.2%)	1537 (30.11%)	1638 (30.43%)	
	>cut off value	171 (61.29%)	3541 (69.38%)	3712 (68.96%)	
Neutrophil count	< cut off value	22 (7.89%)	445 (8.72%)	467 (8.68%)	0.003#
	Normal	223 (79.93%)	4311 (84.46%)	4534 (84.23%)	
	>cut off value	34 (12.19%)	348 (6.82%)	382 (7.1%)	
Lymphocytes/WBC	< cut off value	11 (3.94%)	49 (0.96%)	60 (1.11%)	< 0.001#
	Normal	63 (22.58%)	910 (17.83%)	973 (18.08%)	
	>cut off value	205 (73.48%)	4145 (81.21%)	4350 (80.81%)	
Neutrophils/WBC	< cut off value	209 (74.91%)	4053 (79.41%)	4262 (79.18%)	0.003#
	Normal	59 (21.15%)	976 (19.12%)	1035 (19.23%)	
	>cut off value	11 (3.94%)	75 (1.47%)	86 (1.6%)	

Note:^ Mann-Whitney U test; ^^ T test; # Chi-square test; ## Fisher exact method

*CPB* cardiopulmonary bypass, *BMI* body mass index, STS risk grade: Society of Thoracic Surgeons risk grade, *ALT* alanine transaminase, *AST* aspartate aminotransferase, *ALP* alkaline phosphatase, *WBC* white blood cell

#### Univariate and multivariate analysis of risk factors for nosocomial infection in CHD children

Univariate analysis found that history of cardiac surgery, STS risk grade, delayed sternal closure, BMI < 5th percentile, CPB time, aortic clamping time, lymphocyte counts, lymphocyte/WBC ratio, neutrophil count>cut off value, neutrophil/WBC ratio>cut off value, ALT >cut off value and AST>cut off value of nosocomial infection CHD children were all significantly different with control CHD children.

Multivariate analysis found that STS risk grade (OR 1.38, 95%CI: 1167–1.633; *P* < 0.001), BMI < 5th percentile (OR 1.934, 95%CI: 1.377–2.715; P < 0.001), CPB time (OR 1.018, 95%CI: 1.015–1.022; P < 0.001), lymphocyte/WBC ratio<cut off value (OR 3.818, 95%CI: 1.529–9.533; *P* = 0.004) and AST>cut off value (OR 1.546, 95%CI: 1.119–2.136; *P* = 0.008) were significantly associated with nosocomial infection in CHD children (Table 6).

**Table 6:** Univariate and multivariate logistic regression analysis of risk factors for postoperative infection in children with congenital heart disease

Parameter		Univariate analysis		Multivariate analysis	
		OR (95% CI)	*P*-value	OR (95% CI)	*P* value
Age (days)	Unit = 30	0.998 (0.993,1.003)	0.43		
Gender	Female	1.000			
	Male	0.963 (0.756,1.227)	0.761		
Preterm birth	No	1.000			
	Yes	0.989 (0.237,4.124)	0.988		
History of cardiac surgery	No	1.000			
	Yes	2.741 (2.092,3.59)	< 0.001		
STS risk grade	Unit = 1	2.07 (1.843,2.326)	< 0.001	1.38 (1.167,1.633)	< 0.001
Delayed sternal closure	No	1.000			
	Yes	9.268 (2.774,30.965)	< 0.001		
BMI	5th–95th percentile	1.000			
	< 5th percentile	2.209 (1.671,2.922)	< 0.001	1.934 (1.377,2.715)	< 0.001
	> 95th percentile	0.618 (0.287,1.331)	0.219	0.864 (0.37,2.017)	0.736
CPB time	Unit = 1	1.019 (1.016,1.021)	< 0.001	1.018 (1.015,1.022)	< 0.001
Aortic clamping time	Unit = 1	1.027 (1.024,1.031)	< 0.001		
Lymphocyte count	Normal	1.000			
	< cut off value	4.097 (1.736,9.668)	0.001		
	>cut off value	0.735 (0.571,0.947)	0.017		
Lymphocytes /WBC	Normal	1.000			
	< cut off value	3.243 (1.607,6.543)	0.001	3.818 (1.529,9.533)	0.004
	>cut off value	0.714 (0.534,0.956)	0.024	0.959 (0.658,1.399)	0.829
Neutrophil count	Normal	1.000			
	< cut off value	0.956 (0.61,1.497)	0.843		
	>cut off value	1.889 (1.296,2.754)	0.001		
Neutrophil /WBC	Normal	1.000			
	< cut off value	0.853 (0.634,1.148)	0.295		
	>cut off value	2.426 (1.223,4.814)	0.011		
ALT	Normal	1.000			
	< cut off value	1.338 (0.824,2.171)	0.239		
	>cut off value	3.696 (1.974,6.921)	< 0.001		
AST	Normal	1.000			
	>cut off value	1.911 (1.454,2.512)	< 0.001	1.546 (1.119,2.136)	0.008
ALP	Normal	1.000			
	>cut off value	0.596 (0.081,4.369)	0.61		
Serum creatinine	Normal	1.000			
	< cut off value	0 (0,.)	0.98		
	>cut off value	10.22 (0.923,113.101)	0.058		

Note: *CPB* cardiopulmonary bypass, *BMI* body mass index, STS risk grade: Society of Thoracic Surgeons risk grade, *ALT* alanine transaminase, *AST* aspartate aminotransferase, *ALP* alkaline phosphatase, *WBC* white blood cell

#### Nomograms predicting nosocomial infection risk of CHD children after cardiac surgery

**Fig. 3:**
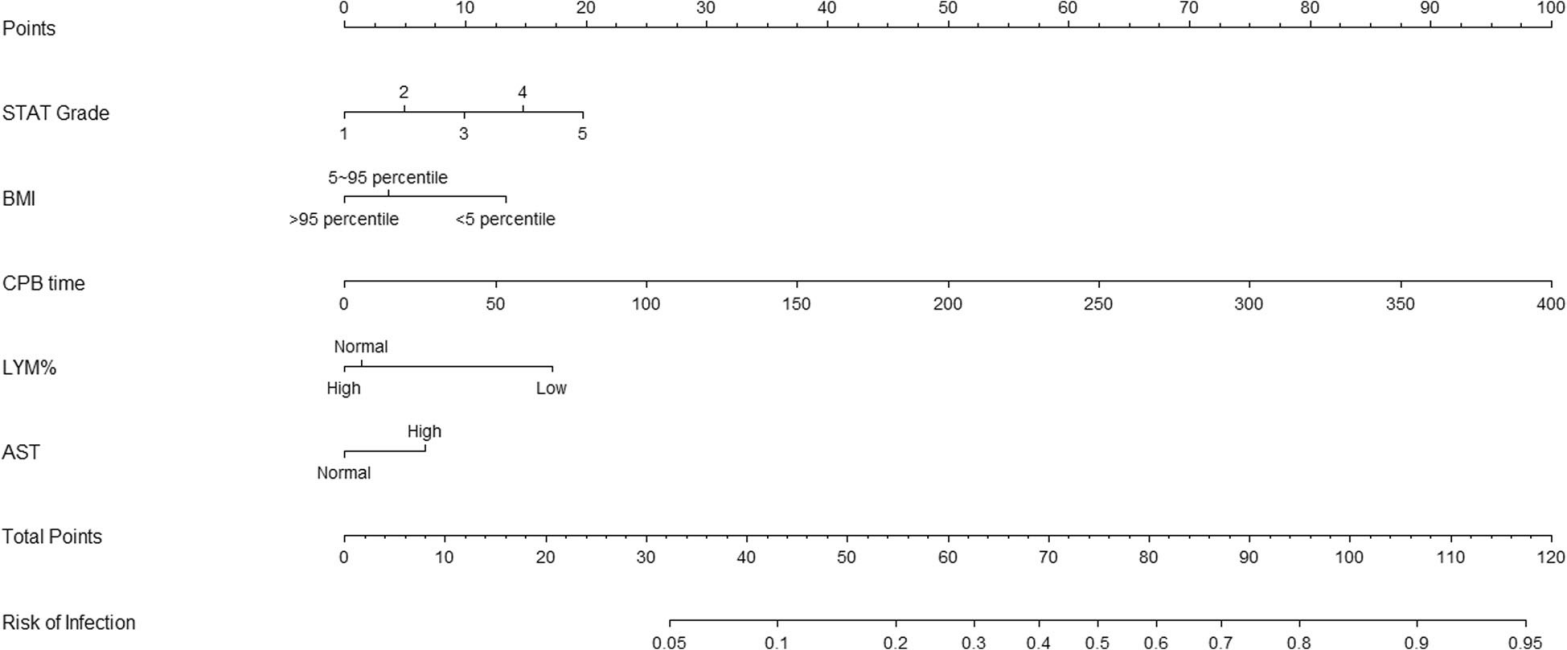
Nomograms predicting nosocomial infection risk of CHD children after cardiac surgery

**Fig. 4:**
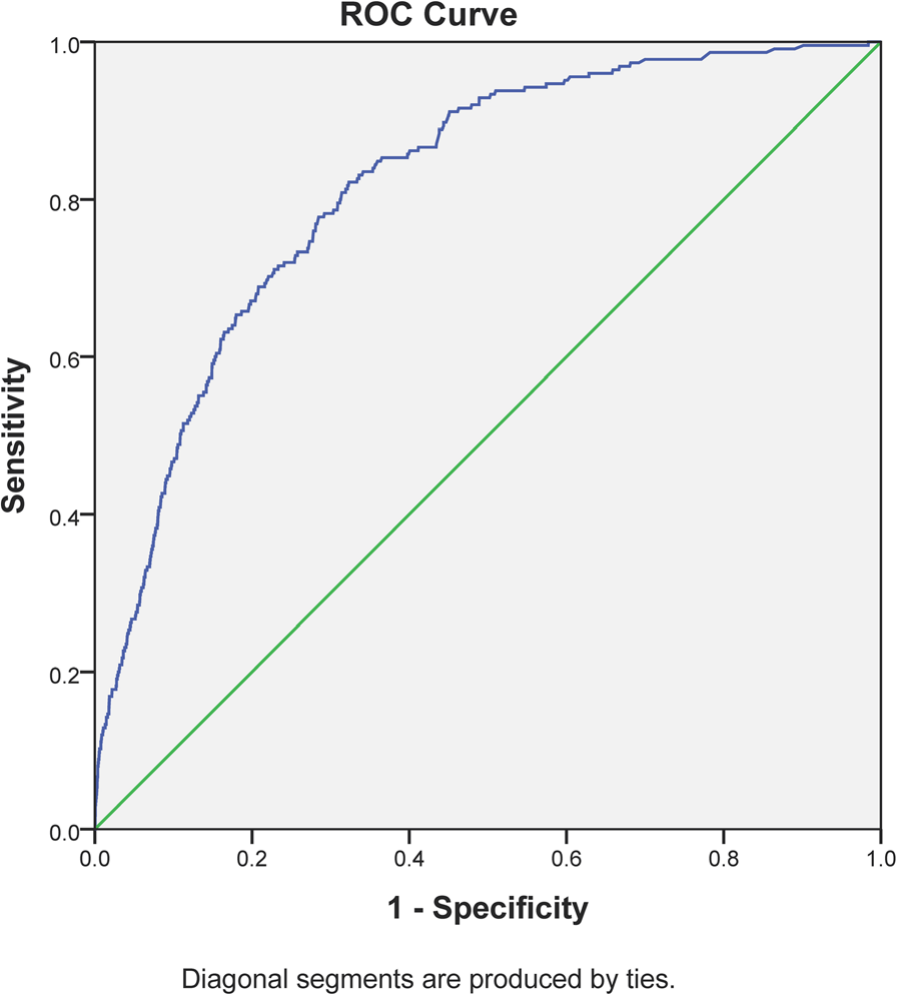
The area under the curve of nomograms predicting nosocomial infection risk of CHD children after cardiac surgery

Nosocomial infection probability of CHD children after cardiac surgery can also be estimated with the nomograms, and calculation method is similar with that of CHD infant (Fig. 3). The AUC of nomograms predicting nosocomial infection risk of CHD children after cardiac surgery was 0.818 (95% CI: 0.792–0.844, *P* < 0.001). After cross validation, AUC of nomograms was 0.808 (Fig. 4).

## Discussion

CHD has become the most common congenital defect in China. Due to China’s large population base, the number of new cases of CHD per year is huge. Surgery is the main treatment for most heart defects [14]. But pediatric cardiac surgery reduces the patient’s immunity and increases the risk of postoperative infection [15]. The study found that nosocomial infection was one of the main complications in the postoperative period in children with CHD, and are major causes of morbidity and mortality after cardiac surgery [16]. Nosocomial infections are also associated with longer intensive care unit (ICU) stay and greater antibiotic usage [17–19]. Analysis and identification of risk factors for nosocomial infections is important for identifying the highest risk population and developing strategies to prevent nosocomial infections. There have been few previous studies on the risk of nosocomial infection after cardiac surgery in children with congenital heart disease, and most of the studies were small size studies. This study investigated the risk factors for infection in 11,937 CHD children after cardiac surgery, the results have important clinical significance for the prevention and treatment of nosocomial infection in CHD children.

In present study, nosocomial infection rate was 10.8%, and nosocomial infection rates of newborns, infant and child were 32.9, 15.4, and 5.2%, respectively. Overall nosocomial infection rate is slightly lower than previous studies [20, 21].

Nosocomial infection rate of CHD newborn was higher than infant and child in our study, age may be one of the main reasons for this difference. We also found that age was significantly associated with nosocomial infection of infant and child after cardiac surgery. Previous studies found that younger age was associated with higher incidence of postoperative infectious complications [22, 23]. Younger age was independently associated with acquisition of bacterial infection post-heart transplantation in children [8]. A matched case-control study found that age younger than 1 year was an independent risk factors for any type of SSI after cardiac surgery in children [24]. Sen et al. assessed the risk factors for postoperative infection after congenital heart surgery using data from the International Quality Improvement Collaborative for Congenital Heart Surgery in Developing World Countries, they found younger age was one of independent risk factors for infection [16].

Surgery itself is the risk of postoperative infection [25]. In our study, STS risk grade and history of cardiac surgery were significantly associated with post-operative nosocomial infection in CHD infant and child. The complexity of surgery is related to the operation time. The more complicated the operation, the longer the operation time, and the worse the outcome of the patient [26]. Duration of surgery and surgical complexity score are all risk factors of nosocomial infections in infants and children undergoing open heart surgery [20, 27]. The possible cause is that the risk of bacterial contamination and cell damage increases at the surgical site as the operation time increases. Retrospective study confirmed high complexity and previous cardiothoracic operation were associated with major infection after pediatric cardiac surgery [25, 28]. A cross-sectional prospective study found that duration of operation ≥3 h significantly predicted surgical site infection [29]. Previous case-control studies showed longer duration of surgery were independently associated with hospitalized surgical site infections after cardiovascular surgery in children [24, 30]. Higher surgical complexity was an independent risk factor for nosocomial infection after congenital heart surgery [16].

Obesity and BMI is associated with an increased number of ventilator days, as well as increased ICU and hospital lengths of stay, which increase the risk of nosocomial infections [31, 32]. Previous study showed that a higher BMI is directly related to longer hospital and ICU stay [33], which increases the risk of nosocomial infections [11, 34]. In a two-center prospective randomized controlled study, multiple regression analysis demonstrated a preoperative BMI of > 30 kg/m2 was an independent predictor for an increased surgical site infection rate after cardiac surgery in adult patients [35]. Higher BMI patients have a higher risk of community acquired and nosocomial infections in the ICU [33, 36, 37]. However, in this study, BMI <5th percentile was significantly associated with increased odds of nosocomial infection after cardiac surgery in CHD infant and child. But BMI >95th percentile was a protective factor for postoperative infection in CHD infant. This correlation is different from that of adults. The possible reason is that the BMI of children with CHD in our study is smaller than general population, and the patients with BMI >95th percentile have not reached the standard of obesity. And BMI <5th percentile may result from CHD and bad nutrition. It also suggested that the better nutrition condition may be helpful to prevent nosocomial infection after cardiac surgery in children. In addition, among the adult population, people with high BMI are prone to diabetes, high blood pressure and other diseases, which are risk factors for postoperative infection [38, 39]. However, children with high BMI have a low risk of developing these diseases. This may also be one of the reasons why higher BMI was associated with decreased odds of postoperative infection risk in CHD children.

During cardiac surgery, CBP induces a systemic inflammatory response that causes immune disorders and significant pulmonary dysfunction [40, 41]. CPB time is closely related to aortic clamping time. Interestingly, we found through multivariate analysis that CPB time was significantly associated with the risk of nosocomial infection after cardiac surgery in children, but there was no significant association with the risk of postoperative infection in infants. The aortic clamping time is just the opposite, and there is only a significant correlation with the risk of postoperative nosocomial infection in infants. Aortic cross-clamp time greater than 85 min was an independent risk factor for surgical site infections in children undergoing cardiac surgery [24]. Lomtadze et al. confirmed that long CPB and cross-clamp time are major risk factors for nosocomial infection after cardiac surgery in a retrospective case study [42].

Neutrophils, the most abundant human immune cells, are rapidly recruited to sites of infection, where they fulfill their life-saving antimicrobial functions [43]. Neutrophils are responsible for nonspecific inflammation through secretion of many inflammatory mediators [44]. This study found neutrophils are involved in the activation of non-specific inflammation, and lymphopenia is associated with adverse outcomes [44]. In our study, high neutrophils/WBC ratio was significantly associated with nosocomial infection in CHD infants, low lymphocyte/WBC ratio was significantly associated with nosocomial infection in child after surgery.

AST is one of the commonly used enzyme indicators for clinical evaluation of myocardial injury, and myocardial damage leads to an increase in AST levels [45]. The patients included in this study were children with congenital heart disease and had varying degrees of myocardial cell damage. These patients have lower immunity than normal people, and the risk of inflammatory reactions and infections increases. This may explain the association between increased AST levels in CHD children and postoperative infection, but further clinical studies are needed.

Combined with the above risk factors, we can use nomogram to assess the risk of nosocomial infections in infants and children with congenital heart disease after cardiac surgery. In our study, the AUC of nomograms predicting nosocomial infection risk of CHD infant after cardiac surgery was 0.738. After cross validation, AUC of nomograms was 0.730. The AUC of nomograms predicting nosocomial infection risk of CHD children was 0.818. After cross validation, AUC of nomograms was 0.808. Therefore, nomograms had good discrimination ability of the risk of nosocomial infection after cardiac surgery in infants and children.

### Limitation

Several limitations should be considered to interpret this study. First, this is a retrospective single-center study. Second, some biomarkers of infection or inflammatory, such as CRP, as well as PCT, was not measured and analyzed. Third, the type of infection such as bacteria or virus, the grams positive of negative bacteria was not analyzed in this study. Fourth, the association between multidrug resistance and postoperative infection cannot be analyzed because of the lack of data related to multiple resistances.

## Conclusion

STS risk grade, BMI, CPB duration, low lymphocyte/WBC ratio or high neutrophil/WBC ratio were independently associated with nosocomial infection in CHD infant and children after cardiac surgery. Additional preventive strategies, including controlling weight (such as nutrition support), optimizing the surgical procedure may reduce risk of postoperative infection, but further research is needed.

## Supplementary information

**Additional file 1: Table S1.** Baseline characteristics of postoperative infection neonates and control.

## Data Availability

The data and materials are not available because of hospital regulation.

## Abbreviations

ALP: Alkaline phosphatase
ALT: Alanine transaminase
ASA: American Society of Anesthesiologist
AST: Aspartate aminotransferase
BMI: Body mass index
CHD: Congenital heart disease
STS risk grade: Society of Thoracic Surgeons risk grade
WBC: White blood cell
