# Supplementary material for: Risk factors of nosocomial infection after cardiac surgery in children with congenital heart disease
Source: BMC Infect Dis. 2020 Jan 21;20:64. doi: 10.1186/s12879-020-4769-6 (PMC6975050; doi:10.1186/s12879-020-4769-6)
Supplement: Supplementary file 1 — Additional file 1: Table S1. Baseline characteristics of postoperative infection neonates and control. [file 12879_2020_4769_MOESM1_ESM.docx]

Table 1. Baseline characteristics of postoperative infection neonates and control

| Parameter |  | postoperative infection (n=28) | control (n=57) | Total (n=85) | P value |
| --- | --- | --- | --- | --- | --- |
| Age (days) | Median (IQR) | 19.5 (11.5,26) | 14 (6,23) | 16 (9,25) | 0.039^ |
| CPB time (min) | Median (IQR) | 85 (51,103) | 90 (68,120) | 90 (61,113) | 0.284^ |
| Aortic clamping time(min) | Median (IQR) | 52 (42,69) | 64.5 (37.5,79) | 59 (38.5,76) | 0.351^ |
| Gender | MALE | 17 (60.71%) | 37 (64.91%) | 54 (63.53%) | 0.705# |
| Preterm birth | YES | 1 (3.57%) | 5 (8.77%) | 6 (7.06%) | 0.659## |
| History of cardiac surgery | YES | 0 (0%) | 0 (0%) | 0 (0%) |  |
| BMI | < 5th percentile | 3 (11.54%) | 3 (5.77%) | 6 (7.69%) | 0.574## |
|  | 5th~95th percentile | 17 (65.38%) | 38 (73.08%) | 55 (70.51%) |  |
|  | > 95th percentile | 6 (23.08%) | 11 (21.15%) | 17 (21.79%) |  |
| STS risk grade | 1 | 0 (0%) | 1 (1.75%) | 1 (1.18%) | 0.354^ |
|  | 2 | 8 (28.57%) | 8 (14.04%) | 16 (18.82%) |  |
|  | 3 | 11 (39.29%) | 27 (47.37%) | 38 (44.71%) |  |
|  | 4 | 9 (32.14%) | 21 (36.84%) | 30 (35.29%) |  |
| Delayed sternal closure | YES | 3 (10.71%) | 3 (5.26%) | 6 (7.06%) | 0.391## |
| ALT | < cut off value | 2 (9.52%) | 10 (19.23%) | 12 (16.44%) | 0.236## |
|  | Normal | 17 (80.95%) | 41 (78.85%) | 58 (79.45%) |  |
|  | ＞cut off value | 2 (9.52%) | 1 (1.92%) | 3 (4.11%) |  |
| AST | Normal | 13 (61.9%) | 25 (48.08%) | 38 (52.05%) | 0.284# |
|  | ＞cut off value | 8 (38.1%) | 27 (51.92%) | 35 (47.95%) |  |
| ALP | Normal | 21 (100%) | 52 (100%) | 73 (100%) |  |
| Serum creatinine | Normal | 19 (95%) | 41 (93.18%) | 60 (93.75%) | >0.999## |
|  | ＞cut off value | 1 (5%) | 3 (6.82%) | 4 (6.25%) |  |
| WBC counts | Normal | 26 (92.86%) | 56 (98.25%) | 82 (96.47%) | 0.251## |
|  | ＞cut off value | 2 (7.14%) | 1 (1.75%) | 3 (3.53%) |  |
| Lymphocyte count | < cut off value | 4 (14.29%) | 12 (21.05%) | 16 (18.82%) | 0.261# |
|  | Normal | 5 (17.86%) | 17 (29.82%) | 22 (25.88%) |  |
|  | ＞cut off value | 19 (67.86%) | 28 (49.12%) | 47 (55.29%) |  |
| Neutrophil count | < cut off value | 2 (7.14%) | 3 (5.26%) | 5 (5.88%) | 0.026## |
|  | Normal | 19 (67.86%) | 23 (40.35%) | 42 (49.41%) |  |
|  | ＞cut off value | 7 (25%) | 31 (54.39%) | 38 (44.71%) |  |
| Lymphocytes/WBC | < cut off value | 12 (42.86%) | 33 (57.89%) | 45 (52.94%) | 0.405# |
|  | Normal | 12 (42.86%) | 19 (33.33%) | 31 (36.47%) |  |
|  | ＞cut off value | 4 (14.29%) | 5 (8.77%) | 9 (10.59%) |  |
| Neutrophils/WBC | < cut off value | 7 (25%) | 7 (12.28%) | 14 (16.47%) | 0.333## |
|  | Normal | 5 (17.86%) | 9 (15.79%) | 14 (16.47%) |  |
|  | ＞cut off value | 16 (57.14%) | 41 (71.93%) | 57 (67.06%) |  |

Note：^ Mann-Whitney U test； ^^ T test； # Chi-square test； ## Fisher exact method

BMI: body mass index, STS risk grade: Society of Thoracic Surgeons risk grade, ALT: alanine transaminase, AST: aspartate aminotransferase, ALP: alkaline phosphatase, WBC: white blood cell
